# Trends in Overweight and Obesity Among Children and Adolescents in China from 1991 to 2015: A Meta-Analysis

**DOI:** 10.3390/ijerph16234656

**Published:** 2019-11-22

**Authors:** Yaru Guo, Xiaojian Yin, Huipan Wu, Xiaojiang Chai, Xiaofang Yang

**Affiliations:** 1 Key Laboratory of Adolescent Health Assessment and Exercise Intervention, Ministry of Education, College of Physical Education & Health, East China Normal University, Shanghai 200241, China; 17321210960@163.com (Y.G.); 15201805219@163.com (X.C.);; 2 College of Economics and Management, Shanghai Institute of Technology, Shanghai 201418, China; 3 Leisure Sports Department of Taiyuan Institute of Technology, Taiyuan 030008, China; huipan-wu@163.com

**Keywords:** Chinese children and adolescents, overweight, obesity, prevalence

## Abstract

This meta-analysis of overweight and obesity (ow/ob) among children and adolescents in China from 1991 to 2015 provides a reference for promoting the healthy development of children and adolescents. The studies were retrieved from the China National Knowledge Infrastructure, Wanfang, and PubMed databases for the period from January 1991 to January 2018. The data were combined and analyzed, and the combined effect magnitude odds ratio and the 95% confidence interval were calculated. Publication bias was determined using Stata/SE12.0. We found that (1) the prevalence of ow/ob increased from 5.0% and 1.7% in 1991–1995 to 11.7% and 6.8% in 2011–2015, respectively, and the overweight rate was the greatest in 2006–2010; (2) from 1991 to 2015, the prevalence of ow/ob was greater in urban areas than in rural areas; (3) compared with girls, boys were more likely to be ow/ob; and (4) the prevalence rates of ow and ob were greater in infancy than in other growth stages, with values of 11.7% and 7.0%, respectively. The prevalence of ow/ob among Chinese children and adolescents showed significant differences based on region, sex, and age. An overall upward trend was observed that decreased slightly from 2011 to 2015.

## 1. Introduction

The global overweight and obesity rates in childhood and adolescence continue to increase [1]. In China, the rapid development of the economy, the improvement in living standards, and changes in lifestyle, physical inactivity, sedentary behavior, and excessive energy intake have all resulted in a rapid increase in overweight and obesity rates among children and adolescents [2,3,4].

The prevalence rates of overweight and obesity in Chinese children continuously increased from 1985 to 2014, and the annual mean increase rate of obesity was the highest in 2010–2014 [5]. The obesity rate of urban children increased rapidly in 1995–2005, with a mean annual increase of 6.9% [6]. The obesity rate in rural areas increased from 0.71% in 1990 to 1.21% in 2006 [7,8]. Most of these obese adolescents have shown varying degrees of decline in self-esteem, accompanied by related mental health problems, such as anxiety, stress, loneliness, and high-risk behavior [9,10]. Of obese adolescents, 75%–80% are still obese after adulthood, during which obesity continues to facilitate the development of other diseases and reduce life expectancy [11,12]. Overweight and obesity, as important factors affecting the physical and mental health of children and adolescents in China, have attracted considerable attention from many fields.

The research on the prevalence rate of overweight and obesity in children and adolescents in China is mostly regional or restricted to certain age groups or specific years. Summaries about different genders, regions, and ages are lacking in the research on prevalence trends of overweight and obesity among Chinese children and adolescents in the last 25 years. These conditions are disadvantageous to the healthy development of the physique of children and adolescents. Therefore, a meta-analysis of overweight and obese children and adolescents in China from 1991 to 2015 can accurately reflect the current situation as well as changes to promote the healthy development of children and adolescents and to provide a reference.

## 2. Materials and Methods

### 2.1. Literature Retrieval Strategy

Our meta-analysis was conducted in accordance with Preferred Reporting Items for Systematic Reviews and Meta-Analyses (PRISMA) guidelines (File S1). The relevant literature on overweight and obesity in Chinese children and adolescents was retrieved from the China National Knowledge Infrastructure (CNKI), Wanfang database, Wip Chinese sci-tech journal full-text database (VIP), and PubMed for the period from January 1991 to January 2018. The database search strategy was formulated around terms “China OR Chinese” AND “infant OR childhood OR children OR toddler OR adolescence OR adolescents OR youth OR teen OR teenager” AND “obesity OR overweight OR body mass index OR BMI OR weight gain” AND “incidence OR frequency OR prevalence OR epidemiology”.

### 2.2. Documentation Inclusion and Exclusion Criteria

#### 2.2.1. Literature Inclusion Criteria

The inclusion criteria were (1) samples with children and adolescents aged 0–18 in China, (2) the number or detection rate of overweight and obesity in the original literature, and (3) the study type was cohort or cross-sectional. Some of the literature used body mass index (BMI = weight/height^2^ (kg/m^2^)) to determine overweight and obesity, including the criteria of the Working Group for Obesity in China (WGOC) [13], World Health Organization (WHO/NCHS) [14], International Obesity Task Force (IOTF) [15], and Centers for Disease Control and Prevention (CDC, USA) [16]. We also included studies that classified overweight and obesity on the basis of ideal weight deviations. The ratio of body weight (W) to ideal weight (IW) was calculated, with W/IW > 1.1 defined as overweight and W/IW > 1.2 defined as obesity [17].

#### 2.2.2. Literature Exclusion Criteria

**Figure 1:**
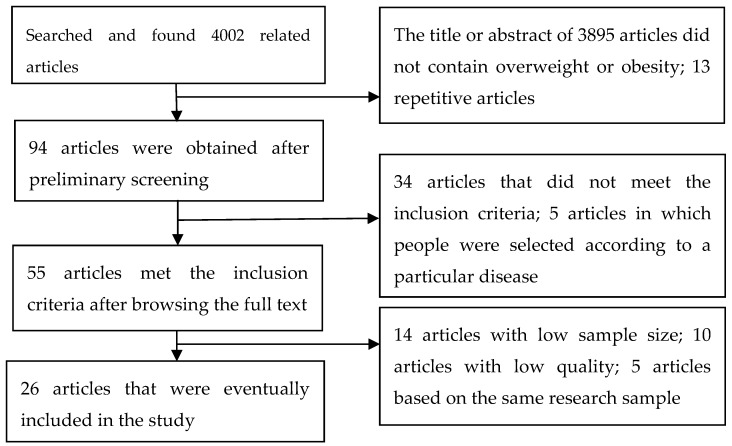
Flowchart of article screening and selection process.

We excluded articles that contained one of the following characteristics: (1) study of non-overweight and non-obese, (2) study on a population selected on the basis of specific diseases, (3) repeated publication and study of a region or population in the same year, (4) a sample size below 1000, and (5) low quality (Figure 1).

### 2.3. Data Extraction

The two researchers independently screened and analyzed the literature according to the inclusion and exclusion criteria, and any differences in opinion were resolved through third-party consultation. The information extracted from the literature mainly included the author, publication year, survey time, research design, representation of the target population, sample selection, sample size, response rate, reasons for non-selection, data sources, data collection, personal and demographic characteristics (obesity, overweight, sex, age, and urban and rural descriptions), epidemic recall period, study objectives, criteria for overweight/obesity, and data collection. The quality assessment extraction form (Table S1) reported by Yu et al. [18] was used to evaluate the quality of the included documents. A score above 14 (the highest is 19 points) is high-quality literature, a score of 11–14 indicates medium-quality literature, and a score below 11 is low-quality literature. Articles with less than 11 points that were classified as low quality, were excluded.

### 2.4. Statistical Analysis

The literature was divided into five periods according to the year of investigation (1991–1995, 1996–2000, 2001–2005, 2006–2010, and 2011–2015) and compared and analyzed according to sex, age, and place of residence (urban and rural). Open-Meta (Agency for Healthcare Research & Quality, Rockville, MD, USA) was used to calculate the positive rate of overweight or obesity by time, sex, age, and residence and the 95% confidence interval (CI). The data were analyzed using Review Manager 5.1.4 (Nordic Cochrane Center, Copenhagen, Denmark), and the combined effect odds ratio (OR) value and 95% CI were calculated. For the heterogeneity test, the test level was α = 0.05. If there was no statistical heterogeneity, the fixed effects model was used; otherwise, the random effects model was used [19]. Sensitivity analysis was used to determine sample size, study quality, diagnostic criteria, geographic distribution, and whether the type of survey affected the research findings. Publication bias was assessed by inspecting funnel plots, and formal testing for funnel plot asymmetry was performed using the Begg and Egger tests, which were conducted using Stata/SE12.0 (Stata Corp., College Station, TX, USA) [20].

## 3. Results

### 3.1. Included Document Characteristics

#### 3.1.1. Literature Search Results

A total of 4002 related studies were found by searching four databases. We included 26 articles (41 studies), of which 14 were from national surveys and 12 were from regional surveys (18 in Chinese and 8 in English).

#### 3.1.2. General Situation and Quality Evaluation of the Literature

In the quality evaluation of the 26 articles, we found 9 high-quality and 17 medium-quality documents (Table 1).

### 3.2. Meta-Analysis Results

#### 3.2.1. Prevalence of Overweight in Children and Adolescents

##### Sex Differences

Irrespective of sex, overweight rates showed an overall upward trend over time, with a slight decline in 2011–2015 (Table S2). Overall, the overweight rate increased from 5.0% in 1991–1995 to 13.2% in 2006–2010, and it dropped to 11.7% in 2011–2015. In 1991–1995, the overweight rates of boys and girls were 5.7% and 4.3%, respectively. The rate peaked in 2006–2010, rising to 16.0% for boys and 10.3% for girls. Between 2011 and 2015, the rates for boys and girls fell to 14.4% and 9.1%, respectively. In the same period, the rate of overweight boys was higher than that of overweight girls.

**Figure 2:**
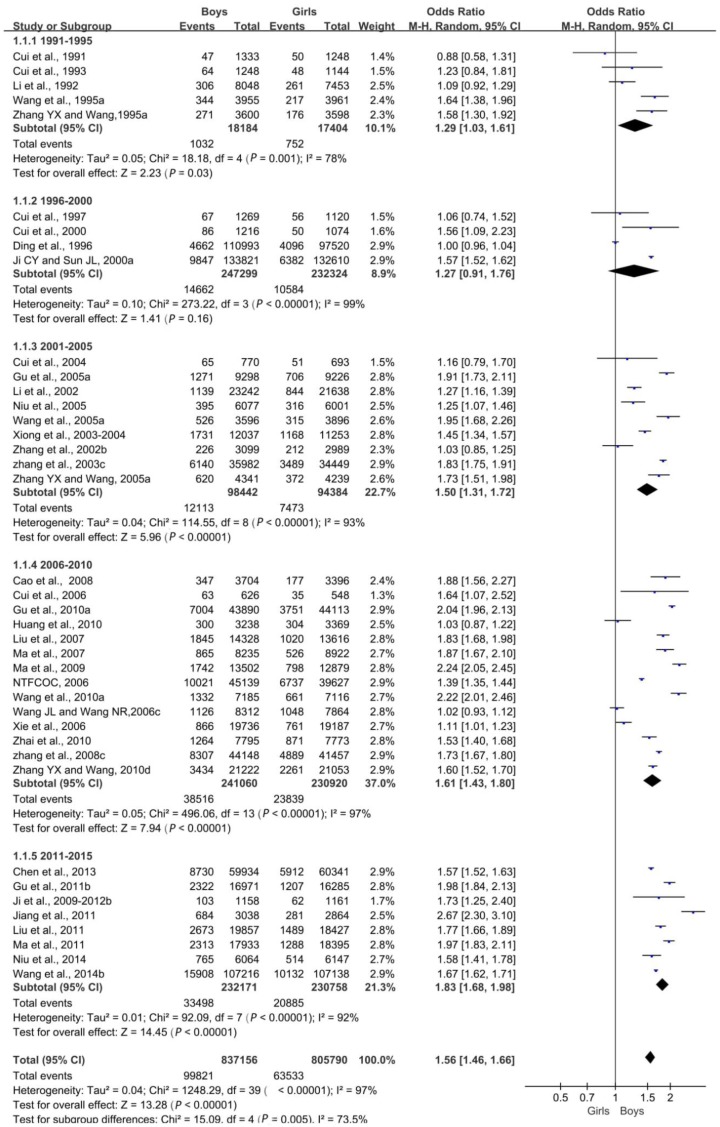
Forest plot of overweight in boys compared with girls aged 0–18 years in China.

An absolute comparison of overweight boys and girls (OR) was performed, and a forest map was drawn. Overall, the difference in the prevalence of overweight was statistically significant (*p* < 0.05); that is, the overweight rate was related to sex, and the sex difference in the detection rate of overweight was increasing each year (Figure 2).

##### Urban and Rural Differences

Overall, both urban and rural overweight rates showed an upward trend over time, with a slight decline in 2011–2015 (Table S3). Among them, the overweight rate of urban children and adolescents reached a peak of 16.1% in 2001–2005 and then decreased. The overweight rate of children and adolescents in rural areas reached a peak of 12.3% in 2006–2010, 3.7 times higher than the peak rate of 3.3% in 1991–1995. In the same period, the detection rate of overweight in urban areas was higher than that in rural areas.

**Figure 3:**
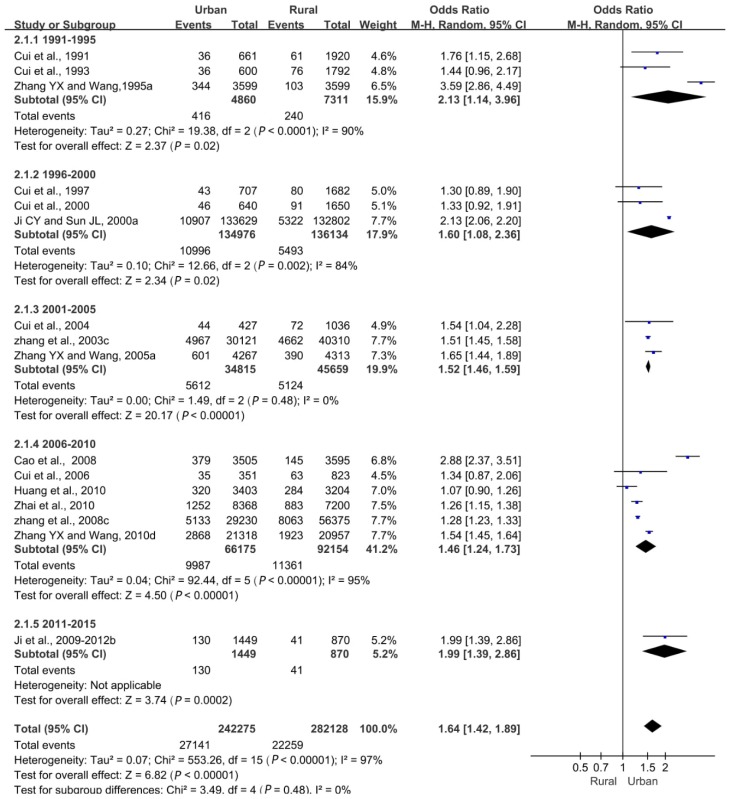
Forest plot of overweight in urban children and adolescents compared with rural children and adolescents aged 0–18 years.

Overall, the difference in the overweight detection rate between urban and rural areas was statistically significant (*p* < 0.05), meaning that a correlation existed between the overweight rate and residence, and the difference between urban and rural areas became smaller (Figure 3).

##### Differences in Growth and Development Stages

The overweight prevalence showed an increasing trend each year in different growth and development stages. In 1996–2000, the overweight detection rates in infants, early childhood, and preschool children were 4.7%, 1.4%, and 1.9%, respectively. Between 2006 and 2010, the overweight rates increased to 19.4%, 17.6%, and 11.0%, respectively. In 1991–1995, the rates of overweight among school-aged children and adolescents were 4.2% and 3.3%, respectively. In 2011–2015, the overweight rates rose to 12.8% and 10.2%, respectively. The detection rate of overweight in different growth and development stages was 11.7% in infancy, followed by 9.7% in school age, 5.6% in preschool age, and 8.2% in adolescence (Table 2).

#### 3.2.2. Prevalence Rate of Obesity in Children and Adolescents

##### Sex Differences

In general, obesity rates were increasing each year for both boys and girls. Overall, the obesity rate increased from 1.7% in 1991–1995 to 6.8% in 2011–2015 (Table S4). In 1991–1995, the obesity rates of boys and girls were 2.0% and 1.3%, respectively; in 2011–2015, it increased to 8.8% and 4.8%, respectively, which represent 4.4-fold and 3.7-fold increases, respectively. In the same period, the obesity rate of boys was higher than that of girls.

**Figure 4:**
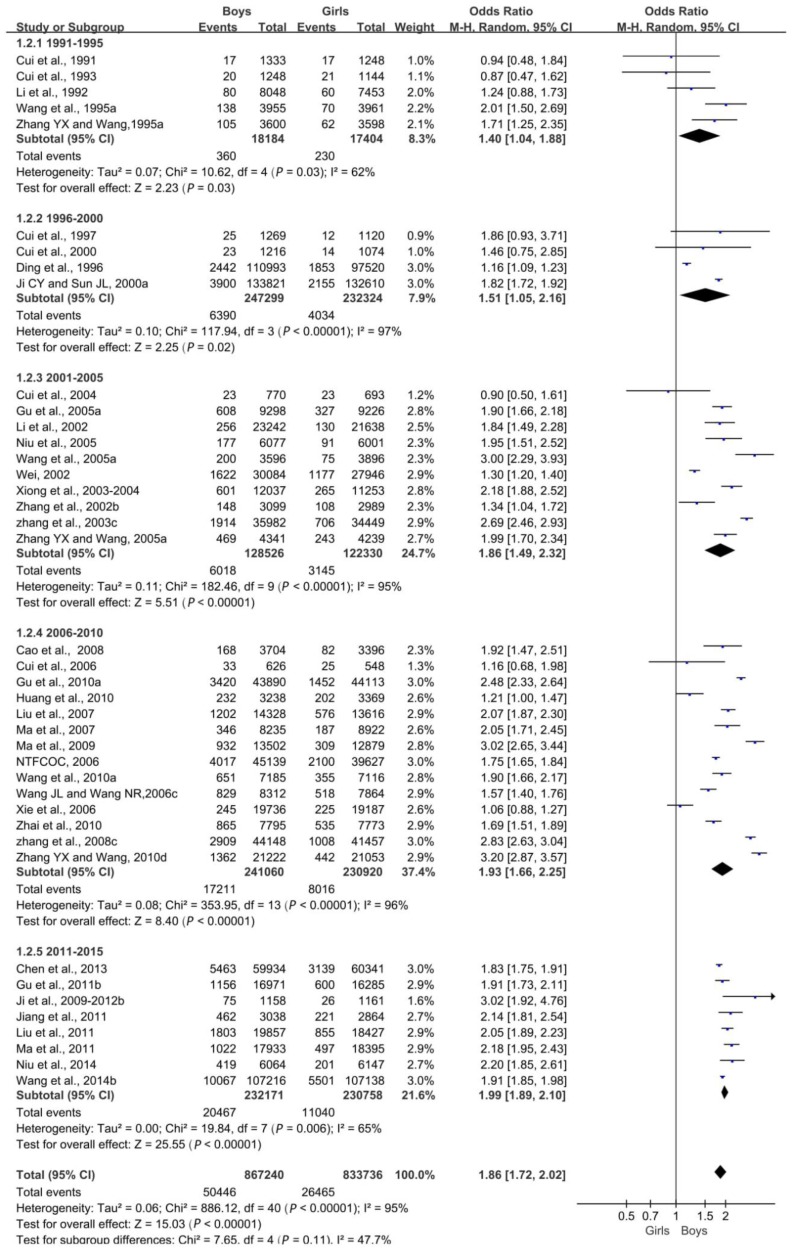
Forest plot of obesity in boys compared with girls aged 0–18 years.

Overall, the difference in the obesity detection rate between boys and girls was statistically significant (*p <* 0.05). The obesity rate was correlated with sex, and the difference between boys and girls was increasingly significant (Figure 4).

##### Urban and Rural Differences

Urban and rural obesity rates generally increased over time, with a slight decline between 2011 and 2015 (Table S5). In 1991–1995, the detection rates of obesity in urban and rural children and adolescents were 3.6% and 0.9%, respectively; in 2006–2010, they reached 7% and 4.5%, respectively; and in 2011–2015, they fell to 5.1% and 3.1%, respectively. Simultaneously, the detection rate of urban obesity was higher than that in rural areas.

**Figure 5:**
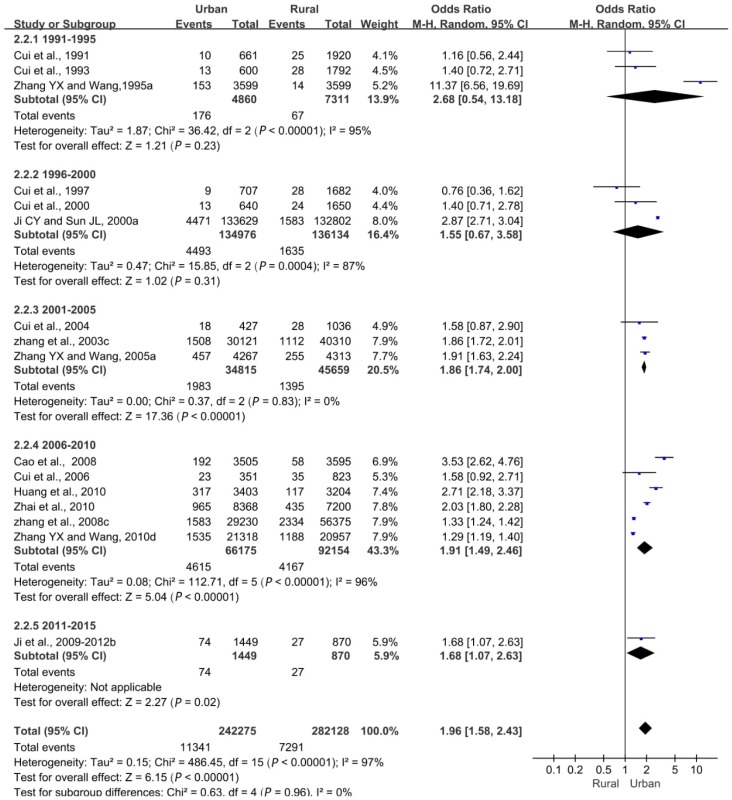
Forest plot of obesity in urban children and adolescents compared with rural children and adolescents aged 0–18 years.

Overall, we found a statistically significant difference in the detection rate of obesity between urban and rural areas (*p* < 0.05). The obesity rate was correlated with the place of residence, and the difference in the detection rate of obesity between urban and rural areas decreased (Figure 5).

##### Differences in Growth and Development Stages

The obesity rate for all growth stages other than infancy showed an increasing trend each year. The infancy obesity rate fell from 8.3% in 1996–2000 to 6.3% in 2006–2010; in toddlers and preschool children, it increased from 4.2% and 3.1% in 1996–2000 to 5.0% and 5.3% in 2006–2010, respectively. Between 1991 and 1995, the obesity rates of school-aged children and adolescents were 1.7% and 0.3%, respectively, and they increased to 8.9% and 3.9% in 2011–2015, respectively. The comparison of obesity detection rates at different growth and development stages revealed a value of 7.0% in infancy, followed by 5.5% during school age and 2.8% in adolescence (Table 3).

### 3.3. Heterogeneity Test and Publication Bias

In this study, the heterogeneity (*I*^2^ > 50%) of the pooled prevalence was high. Sensitivity analysis is an indirect method for analyzing heterogeneity. It mainly re-examines the combined effect by removing certain types of literature and compares the new combined results with the combined results before the exclusion. Sensitivity analysis of the sample size, study quality, diagnostic criteria, geographic distribution, and survey type showed these were important factors in research heterogeneity (Table S6).

**Figure 6:**
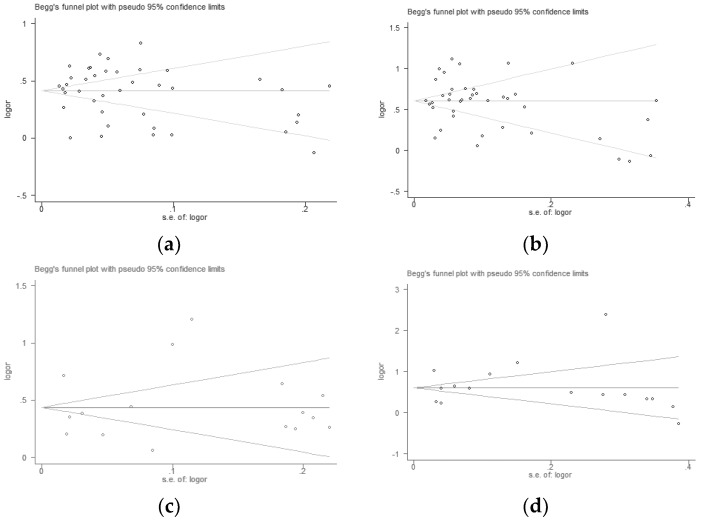
Publication bias in overweight and obesity.

According to the included studies, Begg’s funnel plots were drawn. The Begg’s test and Egger’s test further showed that there was no publication bias in overweight (Figure 6a, Begg’s test, *p =* 0.328, and Egger’s test, *p =* 0.892; Figure 6c, Begg’s test, *p =* 0.392, and Egger’s test, *p =* 0.947) and obesity (Figure 6b, Begg’s test, *p =* 0.159, and Egger’s test, *p =* 0.976; Figure 6d, Begg’s test, *p =* 0.192, and Egger’s test, *p =* 0.930).

## 4. Discussion

From 1991 to 2015, the prevalence rate of overweight and obesity among Chinese children and adolescents increased, but it decreased slightly from 2011 to 2015. In 1991–2000, the overweight and obesity rates increased steadily, and sex differences were not obvious. This may be related to the gradual improvement in living standards and the improved nutrition of children in China after the reform and opening-up at the end of the 1970s. The decrease in the detection rate of overweight and obesity from 2010 to 2015 may be closely related to a series of important sports policies and regulations promulgated by Chinese government agencies. Since the reform and opening-up, the level of nutrition and morphological development of adolescents have continuously improved, and these trends have considerably improved the health of the people as a whole. However, the government departments of our country are aware that the unilateral pursuit of the rate of enrollment has led to schoolwork that is too heavy a burden on students, and rest and exercise time are seriously insufficient. Insufficient sports facilities and conditions create barriers to students’ physical education and sports activities. Physical fitness monitoring showed that physical fitness indicators, such as endurance, strength, and speed, continued to decline in young people; additionally, the rate of poor eyesight was high, and the proportion of urban overweight and obese adolescents increased significantly. The nutritional status of some rural adolescents needs to be improved. Therefore, the government department implemented the Opinions of the Central Committee of the Communist Party of China on Strengthening Youth Sports to Enhance the Physical Fitness of Young People in 2007 [45] while emphasizing the reduction of students’ schoolwork burden, ensuring an hour of exercise every day, and extensively implementing the National Million Students Sunshine Sports. The relevant survey results show that because of the promulgation and implementation of the opinions in 2007, China carried out all-round sunshine sports activities, with 95.3% of the primary school group and 85.7% of children in the middle-school group achieving passing grades for the National Student Physical Health Standard. The effect of sunshine sports is remarkable [46]. The State Sports General Administration formulated the 12th Five-Year Plan according to the overall arrangement of the CPC Central Committee and the State Council, as well as the new situation, new tasks, and new requirements faced by sports development in our country during the 12th Five-Year Plan period. Among them, the recommendation for children and adolescents was to implement the Youth Sports Promotion Program to improve the health quality of adolescents [47]. 

The detection rates of overweight and obesity in boys were higher than those in girls, and the gender difference increased every year, which is consistent with the results reported by Xue et al. in 2014 [48]. This may be related to cognition and dietary and physical activity behavior. For example, the tendency of parents to recognize the differences in the physical development of boys and girls may lead to feeding differences, which mainly manifest in the overfeeding of boys, thereby increasing the rate of overweight and obesity in boys compared with that in girls [49,50]. Girls and boys have different perceptions of weight and diet [51]: girls feel more pressure to be thin and suffer lower self-evaluation [52]; even if their weight is normal, they think that they need to lose weight [53]. Conversely, boys rarely think that they need to lose weight, even if they are overweight. Girls, especially adolescent girls, in pursuit of a slim figure, consciously control their diet and may have unhealthy eating behaviors, such as partial eating, picky eating, and blind dieting [54]. The 2005 risk behavior monitoring report for adolescents noted that 29.1% of boys play computer games for more than two hours a day, 2.0 times higher than girls [55].

The prevalence rates of overweight and obesity in urban children and adolescents were higher than in rural areas, but the difference between urban and rural areas decreased every year, which is consistent with the results reported by Song et al. [56]. In China, more overweight and obese children and adolescents come from economically wealthy families and have parents with higher education, and the role of socioeconomic status (SES) is opposite to that in developed countries [57,58,59,60,61]. This may be the result of higher SES requirements for children, whose weekly intake of meat or fish, eggs, dairy products, beans, and fruits and vegetables in urban areas is significantly higher than that of children in rural areas, resulting in overweight urban children and adolescents. The prevalence of obesity in urban areas was much higher than that in rural areas [62]. Second, the lack of physical activity time and the increase in the stationary behavior of urban children and adolescents may also lead to overweight urban children and adolescents [2]. Finally, exposure to high concentrations of air pollution was positively correlated with overweight and obesity in 2–13-year-old children [63]. Therefore, air pollution is a potential factor of the higher rate of overweight and obese children and adolescents in urban areas compared with those in rural areas. Zhang et al. [64] pointed out that between 1985 and 2014, the prevalence of overweight and obesity among rural children in Shandong province increased rapidly, and the problem of overweight and obesity in rural areas cannot be ignored. Simultaneously, China’s annual growth rate of urbanization increased from 0.53% in 1991 to 1.61% in 2010 [65], consistent with the increasing trend of the rate of overweight and obese children and adolescents. This increase in urbanization may be the main factor of the gradually decreasing difference between urban and rural areas.

Overall, compared with other growth and development stages, the prevalence of overweight and obesity was the highest in infants, which is consistent with the results of Chen et al. [66]. This may be related to the rapid proliferation of adipose tissue in infants and young children in the d [67]. High energy intake in the third trimester of pregnancy and passive feeding might also have led to the highest prevalence of overweight and obesity occurring in infants [68]. In China, many parents prefer to compare the weight of their children with those of other infants, mistakenly believing that heavier children are healthier [69]. On a global scale, the rate of overweight and obesity among children under five years of age increased sharply during the period of 1990–2010, and it increased the most in low- and middle-income countries [70]. In the Iranian region, children over the age of 2–6 and 7–11 years have a higher overweight trend than the older group [71]. This is consistent with the relatively low rate of overweight and obesity in adolescence in this study. With the rapid development of the economy, parents are more likely than in the past to purchase high-fat, high-energy food, and such diets can cause children’s rapid weight gain. The high obesity rate of school-aged children may be related to their poor self-control, parents’ fear of providing insufficient food and thus overfeeding, and less exercise. The reason for the decline in the detection rate during adolescence may be that with the increase in age, the health consciousness of adolescents and that of their parents strengthens, the demands of body shape improve, and the regulation of exercise and diet tightens [72]. The birth cohort of preschool children in 2001–2005 has highest through ages of 4-18 years (preschool to adolescence), but it has a prevalence rate smaller than other birth cohorts when they were infants or toddlers. It may be caused from a publication bias, or use of different definition.

The key to preventing adolescent and even adult obesity is the first year of life [36]. Foreign and domestic studies have shown that breastfeeding can reduce the risk of obesity in children [73,74]. Therefore, health education should be improved to reduce the prevalence of overweight and obesity in infants. We should simultaneously strengthen primary and middle-school children’s awareness of the risk of becoming overweight and obese, continue to implement and improve relevant policies, and increase the level of physical activity of children and adolescents to effectively reduce the detection rate of overweight and obesity among children and adolescents. Studies have shown that interventions targeting healthy eating in children may have a greater impact if healthful foods are made available and easily accessible in the home and if these healthful foods are also consumed by mothers or other family members in the household [75]. Therefore, parents should receive nutrition education to help young children develop healthy eating habits and thus improve the overall growth and development of children while preventing obesity.

Sensitivity analysis found that differences in sample size, study quality, diagnostic criteria, geographic distribution, and survey type were the reasons for the heterogeneity between studies. The Begg test results were not significant and the funnel plot did not show significant publication bias effects. However, because the included studies may be based on one or two of the three classes of gender, region and age, we are unable to obtain all levels of relevant data in the same sample, which gives us the inconvenience of conducting nine levels of research in the same sample. Therefore, this study investigated the prevalence of overweight and obesity by gender, region and age, respectively, which may introduce a bias.

## 5. Study Limitations

To date, the criteria for dividing overweight and obesity have not been unified. Therefore, during data collection, we classified overweight and obesity according to the division method in the literature, so some errors occurred that did not affect the general trend. However, we hope that all regions and organizations will be able to unify standards when conducting research so that they can be accurately compared.

Regional differences exist in overweight and obesity among children and adolescents in China. We did not divide the results by region. In the future, follow-up studies should be conducted on the rates of overweight and obesity in different regions. Different regions vary in terms of dimensions, elevation, living habits, eating habits, and economic conditions.

In a sensitivity analysis, the sample size, study quality, diagnostic criteria, geographic distribution, and type of survey are all important factors that contribute to research heterogeneity. For example, the size of the sample will affect the weight of the overweight and obesity detection rate; the quality of the study will affect the reliability of the detection rate of overweight and obesity; the difference in diagnostic criteria will lead to differences in the detection rate of overweight and obesity. These factors may lead to the underestimation or overestimation of the problem of overweight and obesity in Chinese children.

## 6. Conclusions

The meta-analysis of overweight and obesity among Chinese children and adolescents for the period from 1991 to 2015 showed significant differences in the prevalence of overweight and obesity based on region, sex, and age. The overall trend was upward, but it decreased slightly from 2011 to 2015.

According to the actual situation of local children and adolescents, each region should formulate corresponding prevention and control strategies and pay special attention to high-risk groups.

## Figures and Tables

**Table 1:** General information and quality ratings included in the literature from 1991 to 2015.

Author, Year	Time Period	Sample Size	Age (Years)	Criteria	Target Population	Grade	Overweight, Prevalence, %	Obesity, Prevalence, %
**National Survey on Childhood Obesity (NSCO)**								
Ding et al., 1998 [21]	1996	208,513	0–7	NCHS/CDC	Urban children	15	4.2	2.1
NTFCOC, 2008 [22]	2006	84,766	0–7	W/IW (WHO)	Urban children	15	19.8	7.2
Xie et al., 2014 [8]	2006	38,923	3–7	BMI(WHO)	Rural	13	4.2	1.2
Cao et al., 2011 [23]	2008	7100	7–18	BMI (WGOC)	Urban/rural	15	7.4	3.5
Chen et al., 2017 [24]	2013	120,275	7–18	BMI (WGOC)	Urban/rural	16	12.2	7.2
**China Health and Nutrition Survey (CHNS)**								
Cui et al., 2010 [25]	1991/1993/1997/ 2000/2004/2006	2581/2392/2389/ 2290/1463/1174	7–17	BMI (WGOC)	Urban/rural	13	3.8/4.7/5.1/ 5.9/7.9/8.3	1.4/1.7/1.6/ 1.6/3.1/4.9
**Chinese National Nutrition and Health Survey (CNNHS)**								
Li et al., 2008 [26]	1992/2002	15,501/44,880	7–17	BMI(WHO)	Urban/rural	17	3.7/4.4	0.9/0.9
**Chinese National Survey on Children Constitution and Health (CNSCCH)**								
Wang et al., 2017a [27]	1995/2005/2010	7916/7492/14,301	7–18	BMI (WGOC)	Not reported	14	7.1/11.2/13.9	2.6/3.7/7.0
Gu et al., 2017a [28]	2005/2010	18,524/21,701	7–18	BMI (WGOC)	Urban/rural	14	10.7/12.2	5.0/5.5
Huang et al., 2012 [29]	2010	6607	7–18	BMI (WGOC)	Urban/rural	15	9.1	6.6
Ji CY and Sun JL, 2005a [30]	2000	266,431	7–18	BMI (WGOC)	Urban/rural	17	6.1	2.3
Wang et al., 2017b [5]	2014	214,354	7–18	BMI (WGOC)	Urban/rural	17	12.1	7.3
Zhang YX and Wang SR, 2008a [31]	1995/2005	7198/8580	7–18	BMI (WGOC)	Urban/rural	15	6.2/11.6	2.3/8.3
Zhai et al., 2017 [32]	2010	15,568	7–18	BMI (WGOC)	Urban/rural	16	13.7	9.0
**Regional Survey on Childhood Obesity (RSCO)**								
Zhang et al., 2003b [33]	2002	6088	1–7	W/IW(WHO)	Urban/rural	12	7.2	4.2
Wei, 2007 [34]	2002	58,030	0–6	W/IW(WHO)	Urban	12		4.8
Xiong et al., 2005 [35]	2004	23,292	3–18	BMI (IOTF)	Urban	12	12.4	3.7
Wang JL and Wang NR, 2008c [36]	2006	18,320	0–18	BMI (CDC)	Urban	12	13.4	8.3
Zhang et al., 2012c [37]	2003/2008	70,431/85,605	6–18	BMI (IOTF)	Rurban	12	13.7/15.4	3.7/4.6
Niu et al., 2016 [38]	2005/2014	4956/5308	7–18	BMI (WGOC)	Urban/rural	14	5.9/10.5	2.2/5.1
Liu et al., 2014 [39]	2007/2011	27,944/38,284	5–18	BMI (WGOC)	Not reported	11	10.3/10.9	6.4/6.9
Ji et al., 2016b [40]	2009-2012	2319	6–17	BMI(WHO/WGOC)	Urban/rural	11	7.1	4.4
Gu et al., 2013b [41]	2011	33,256	7–18	BMI (WGOC)	Not reported	12	10.6	5.3
Jiang et al., 2014 [42]	2011	5902	8–15	BMI (IOTF)	Not reported	11	16.4	11.6
Ma et al., 2014 [43]	2007/2009/2011	17,157/26,381/36,328	12–18	BMI (WGOC)	Not reported	12	8.1/9.6/9.9	3.1/4.7/4.2
Zhang YX and Wang SR, 2013d [44]	2010	42,275	7–18	BMI (IOTF)	Urban/rural	11	13.5	4.3

**Table 2:** Subgroup analysis by development stage of the prevalence of overweight in Chinese children and adolescents aged 0–18 years (%).

Time Period	Infancy (Age 0–1 Years)	Toddlers (Age 1–3 Years)	Preschool Children (Age 4–6 Years)	School Children (Age 7–13 Years)	Adolescents (Age 14–18 Years)
1991–1995	-	-	-	4.2 (3.8–4.5)	3.3 (2.9–3.7)
1996–2000	4.7 (4.5–5)	1.4 (1.3–1.5)	1.9 (1.8–2.0)	6.0 (5.9–6.2)	6.3 (6.1–6.4)
2001–2005	-	9.7 (8.1–11.2)	11.8 (10.8–12.9)	6.6 (6.4–6.8)	7.1 (6.8–7.5)
2006–2010	19.4 (18.9–19.9)	17.6 (17.3–18)	11.0 (10.7–11.2)	13.4 (13.2–13.7)	9.1 (8.9–9.3)
2011–2015	-	-	7.5 (6.5–8.5)	12.8 (12.6–13)	10.2 (10.0–10.4)
Total	11.7 (11.4–11.9)	7.4 (7.2–7.5)	5.6 (5.5–5.7)	9.7 (9.6–9.7)	8.2 (8.1–8.3)

**Table 3:** Subgroup analysis by development stage of the prevalence of obesity in Chinese children and adolescents aged 0–18 years (%).

Time Period	Infancy	Toddlers	Preschool Children	School Children	Adolescents
1991–1995	-	-	-	1.7 (1.4–1.9)	0.3 (0.2–0.4)
1996–2000	8.3 (8.0–8.7)	4.2 (4.0–4.3)	3.1 (3.0–3.2)	2.8 (2.7–2.9)	1.8 (1.7–1.8)
2001–2005	4.5 (4.1–5.0)	2.1 (1.9–2.2)	6.6 (6.4–6.9)	1.8 (1.7–1.9)	1.6 (1.4–1.8)
2006–2010	6.3 (5.9–6.6)	5.0 (4.8–5.2)	5.3 (5.1–5.4)	7.5 (7.3–7.7)	3.5 (3.4–3.6)
2011–2015	-	-	11.3 (10.0–12.5)	8.9 (8.7–9.1)	3.9 (3.7–4.0)
Total	7.0 (6.8–7.2)	4.1 (4.0–4.2)	4.5 (4.4–4.6)	5.5 (5.4–5.6)	2.8 (2.7–2.9)

## Supplementary Materials

The following are available online at https://www.mdpi.com/1660-4601/16/23/4656/s1, Table S1: Summary of studies reporting the prevalence of overweight in Chinese children and adolescents aged 0–18 years; Table S2: Summary of studies and the reported prevalence of being overweight in urban and rural children and adolescents age 0-18 years; Table S3: Summary of studies reporting the prevalence of obesity in Chinese children and adolescents aged 0–18 years; Table S4: Summary of studies and their reported prevalence of obesity in urban/rural children and adolescents aged 0–18 years; Table S5: Summary of studies and their reported prevalence of obesity in urban/rural children and adolescents age 0–18 years; Table S6: Sensitivity analysis of the studies on the prevalence of overweight/obesity in children and adolescents; File S1: PRISMA 2009 checklist.doc.
