# Supplementary material for: Trends in Overweight and Obesity Among Children and Adolescents in China from 1991 to 2015: A Meta-Analysis
Source: Int J Environ Res Public Health. 2019 Nov 22;16(23):4656. doi: 10.3390/ijerph16234656 (PMC6926698; doi:10.3390/ijerph16234656)
Supplement: Supplementary file 1 [file ijerph-16-04656-s001.zip › Appendix/Table S1.doc]

| **TableS1.** Quality-assessment extraction form. | |
| --- | --- |
| **Assessment items** | **Score** |
| **1. Study design** |  |
| Prospective | 2 |
| Retrospective | 1 |
| No information | 0 |
| **2. Representativeness of target population** |  |
| Entire target population with nationally representative samples | 2 |
| An entire state or province | 1 |
| A specified number of sampling units (i.e., cities or towns, clinics) | 0 |
| **3. Sample selection** |  |
| Multistage, stratified, random sampling | 2 |
| Two- or one-stage, stratified, random sampling | 1 |
| Non-random sampling | 0 |
| **4. Sample size** |  |
| >10,000 | 2 |
| 5,000–10,000 | 1 |
| <5,000 | 0 |
| **5. Response rate** |  |
| >90% | 2 |
| 70–90% | 1 |
| <70% | 0 |
| **6. Reasons for nonresponse** |  |
| Detailed description | 2 |
| Unclear description | 1 |
| No information | 0 |
| **7. Data source and study objectives** |  |
| Primarily from a national study on prevalence of overweight/obesity | 2 |
| Primarily from a state or provincial study on prevalence of overweight/obesity | 1 |
| Primarily from a study on prevalence of overweight/obesity in small sampling units (i.e., cities or towns, clinics) or a survey not specifically designed for the study of the prevalence of overweight/obesity | 0 |
|  |  |
| **8. Data collection** |  |
| Timely measurement and recording | 2 |
| A validated questionnaire/interview | 1 |
| Retrospectively from records | 0 |
| **9. Description of obesity, overweight, sex, age, urban and rural** |  |
| All points | 2 |
| 3–4 points | 1 |
| 1–2 points | 0 |
| **10. Prevalence recall periods** |  |
| Stated | 1 |
| No information | 0 |
| Interpretation: score >14 (maximum, 19), high quality; 11–14, medium quality; <11, low quality | |
